# The Utility of Gallium Scan in Patients With Perinephric Abscess and Septic Pulmonary Emboli in the Absence of Right-Sided Infective Endocarditis: A Case Report

**DOI:** 10.1155/crdi/2319787

**Published:** 2025-09-17

**Authors:** Bahy Abofrekha, Mohammad Aldalahmeh, Dillon Rogando, Omar Abureesh, Georges Khattar, Neville Mobarakai

**Affiliations:** ^1^Internal Medicine Department, Northwell Health, New Hyde Park, New York, USA; ^2^Infectious Disease Department, Northwell Health, New Hyde Park, New York, USA

**Keywords:** cavitary lesions, CT-guided drainage, disseminated septic embolism, lung abscess, renal abscess, septic pulmonary emboli

## Abstract

A 43-year-old male with newly diagnosed diabetes developed methicillin-sensitive *Staphylococcus aureus* (MSSA) bacteremia following a foot injury. Despite appropriate antibiotic treatment, the patient's blood cultures remained persistently positive, and imaging revealed septic pulmonary emboli. Both transthoracic and transesophageal echocardiography showed no evidence of right-sided infective endocarditis. A gallium scan, followed by CT and MRI, identified a perinephric abscess as the source of infection. After drainage of the abscess and prolonged antibiotic therapy, the bacteremia was resolved, and pulmonary septic foci were reduced. This case highlights the importance of considering extracardiac sources, such as perinephric abscesses, in cases of persistent bacteremia and septic pulmonary emboli, especially when there is no evidence of endocarditis. And the remarkable utility of gallium scans to detect hidden infections.

## 1. Introduction

*Staphylococcus aureus* bacteremia (SAB) is a significant cause of morbidity and mortality in hospitalized patients, with an estimated incidence of 65 per 100,000 persons per year [1]. In up to one-third of patients with SAB, the primary source of infection cannot be identified [2]. Patients with risk factors such as intravenous drug use, hemodialysis, diabetes, or pre-existing heart disease are particularly susceptible to developing SAB and its associated complications [3]. Complicating the diagnosis, nearly one-third of individuals with gram-positive bacteremia and metastatic disease may not show localizing signs or symptoms [2].

The development of complicated infections like infective endocarditis and distant septic metastatic sites—such as pulmonary septic emboli, splenic lesions, and musculoskeletal foci—significantly increases mortality rates and often necessitates extended treatment with intravenous antibiotics and potentially invasive surgical interventions [2, 4, 5]. A significant gap in clinical practice is the timely diagnosis of specific infectious sources like a perinephric abscess, which is particularly challenging due to its insidious onset, nonspecific symptoms, and slow progression, often leading to diagnostic delays [6]. Most perinephric abscesses arise from gram-negative organisms like *Escherichia coli* or *Klebsiella* species, making *Staphylococcus aureus* a rare etiology [7].

A high index of suspicion is crucial for detecting and treating developing septic foci, whether primary or secondary, especially in high-risk patient populations. Multiple tools, including sonography, computed tomography (CT), magnetic resonance imaging (MRI), and nuclear medicine techniques, are available to help detect these primary and secondary foci.

This case report presents an unusual etiology of septic pulmonary emboli and persistent SAB originating from a perinephric abscess in the absence of infective endocarditis. By sharing this case, we aim to encourage clinicians to consider extracardiac sources when faced with similar clinical scenarios, especially when initial imaging is negative for endocarditis [2, 8, 9].

## 2. Case Presentation

A 43-year-old Hispanic male with no significant past medical history presented to the emergency department with right foot pain three days after stepping on a construction nail. The initial examination was unremarkable, and an X-ray of the right foot revealed no retained foreign body or signs of osteomyelitis. The patient was discharged with a 10-day course of ciprofloxacin 750 mg and griseofulvin 500 mg twice daily.

Four days later, the patient returned, unable to bear weight on the injured foot, and admitted to having never consumed the prescribed oral antibiotics. On arrival, he was hypotensive (BP 99/63 mmHg), tachycardic (130 BPM), and afebrile. Physical examination revealed tenderness over the plantar aspect of the right midfoot with a small, healed puncture wound. His neurological exam was unremarkable.

**Figure 1:**
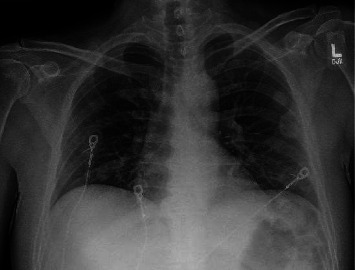
Chest X-ray showing two nodular densities measuring 2.7 and 1.6 cm in the left lung, overlying the fifth and sixth ribs, and scapula.

Initial workup revealed:

• ECG showed sinus tachycardia.
• Chest X-ray showed two nodular densities (Figure 1).
• Elevated lactate (2.6) and white blood cell count (22,000 per microliter) with neutrophilic predominance.
• Blood cultures positive for SAB, specifically methicillin-sensitive *S. aureus* (MSSA).
• Concurrent diabetic ketoacidosis (DKA).

The patient was admitted to the intensive care unit for management of DKA with goal-directed fluid resuscitation and an insulin drip. Over 24 h, his DKA resolved, and he was transitioned to a subcutaneous insulin regimen.

**Figure 2:**
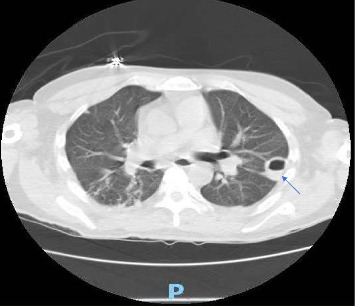
CT showing multiple cavitary lesions, opacities/nodules with peribronchial thickening. Suspicious for infection versus malignancy.

On Day 1 of hospitalization, the patient underwent incision and drainage of the right foot, which yielded only a small amount of phlegmonous material. Tissue cultures grew pan-sensitive *Klebsiella pneumoniae*. Cefazolin 2 gm every 8 h was initiated for the MSSA bacteremia. A CT scan of the chest revealed multiple cavitary lesions (Figure 2), coinciding with the chest X-ray findings and suspicious for right-sided infective endocarditis as a potential source of septic emboli.

On Day 2, transthoracic (TTE) and transesophageal (TEE) echocardiograms were performed, revealing no vegetations or other evidence of endocarditis. Despite 6 days of appropriate antibiotic therapy, blood cultures remained persistently positive for MSSA daily from the second day to the eighth day. Notably, urine cultures were also positive for MSSA. Clindamycin was then added at a dose of 900 mg every 8 h. On the subsequent day (Day 9), the first negative blood culture was obtained.

**Figure 3:**
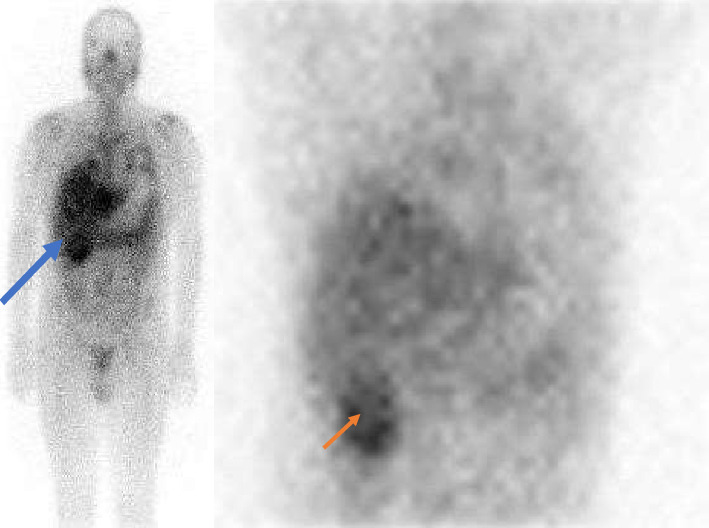
Increased focal uptake in the lungs corresponding to cavitary lesion on CT scan (blue arrow) uptake in the right upper quadrant corresponding to the perinephric collection (orange arrow).

**Figure 4:**
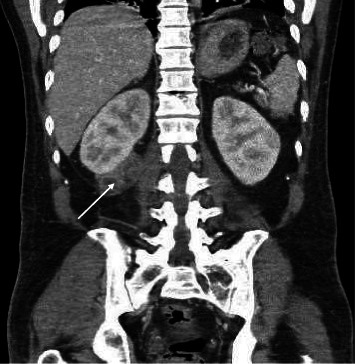
CT scan showing 4.9 cm cystic exophytic lesion in the anterior aspect of the inferior renal pole.

**Figure 5:**
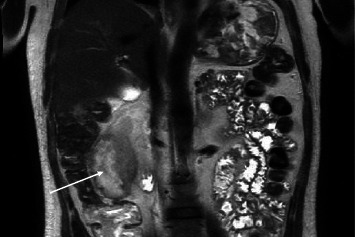
Magnetic resonance imaging of the abdomen showing the perirenal abscess (white arrow).

On Day 9, a gallium scan revealed high uptake in the lung fields and right kidney (Figure 3). A subsequent CT on Day 11 (Figure 4) and MRI on Day 13 (Figure 5) confirmed multiple collections: a 4.8 cm thick-walled abscess in the anterior right kidney, a 3.6 cm complex abscess in the right lower pole, and a 3 cm periprostatic abscess. These occult lesions explained the persistent bacteremia despite adequate sensitivity-guided antibiotic therapy.

**Figure 6:**
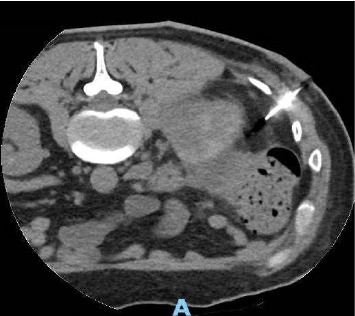
CT scan guided needle drainage of the perinephric abscess.

On Day 14, the largest perinephric abscess was drained percutaneously under CT guidance (Figure 6). The culture of 25 mL of purulent fluid grew MSSA, confirming it as the source of the persistent bacteremia. The periprostatic abscess was not drained due to its small size and anatomically challenging location.

**Figure 7:**
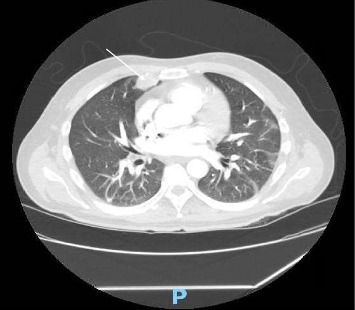
CT scans indicate that the cavitary lung lesions from previous scans have solidified and healed. (white arrow).

After completing a 6-week course of intravenous antibiotics, a repeat chest CT showed resolution of the septic cavitations (Figure 7), and a repeat TTE remained negative for endocarditis.

## 3. Discussion

This case presents an unusual scenario of MSSA bacteremia with pulmonary septic emboli originating from a perinephric abscess without evidence of infective endocarditis. While MSSA is a common pathogen, septic pulmonary emboli without endocarditis are rare [10]. The patient's newly diagnosed diabetes mellitus was a significant risk factor, as it is known to increase the risk of severe disseminated MSSA disease, likely due to impaired innate immune responses [11]. The initial foot wound culture grew *Klebsiella pneumoniae*, while blood cultures consistently yielded MSSA. Despite this, the puncture wound was suspected to be the portal of entry for MSSA, which became invasive and lead to bacteremia. In the literature, common sources for such disseminated infections include osteomyelitis [12], arteriovenous dialysis fistulas [13], and intravenous drug abuse.

The patient's persistent bacteremia, despite appropriate antibiotics, was a key indicator of a metastatic infection and prompted a search for a hidden source [14]. Right-sided infective endocarditis is the most common cause of septic lung emboli from MSSA bacteremia [14, 15]. However, with negative echocardiograms, the investigation correctly shifted to potential extracardiac sources. The positive MSSA urine cultures were an important clue, pointing toward significant renal or urogenital involvement [16].

The use of Gallium-67 (67 Ga) scintigraphy proved crucial. While newer nuclear imaging techniques like 18F-fluorodeoxyglucose positron emission tomography (18F-FDG-PET/CT) are often favored for their speed and sensitivity, the decision was guided by practical considerations at our center. The 67 Ga scan was readily available, whereas obtaining an FDG-PET/CT would have incurred a significant diagnostic delay. This logistical reality, combined with the high specificity of gallium for bacterial infection, made it the optimal choice for this patient. Its mechanism, which involves binding to transferrin and lactoferrin at sites of inflammation, gives it this high specificity [17]. In this case, the 67 Ga scan accurately localized the occult infectious foci, guiding targeted imaging and intervention.

Clinical resolution followed the drainage of the perinephric abscess, with subsequent clearance of the bacteremia and consolidation of the pulmonary abscesses. This outcome underscores the aggressive nature of MSSA organ seeding in diabetic patients and affirms the diagnosis. A similar case by Jung et al. also described a renal abscess as a source of septic pulmonary emboli [18].

## 4. Conclusion

This case report emphasizes the importance of considering extracardiac sources, such as perinephric abscesses, in cases of persistent MSSA bacteremia with disseminated infections, especially when standard evaluations are unrevealing. The early identification of occult infectious foci can lead to lifesaving interventions. Clinicians should maintain a high index of suspicion and utilize advanced imaging techniques when faced with similar clinical scenarios.
